# Nutritional health status: association of stunted and wasted children and their mothers

**DOI:** 10.1186/s12887-022-03309-y

**Published:** 2022-05-06

**Authors:** Ulfat Khadija, Shahid Mahmood, Ammara Ainee, Muhammad Yousaf Quddoos, Hajra Ahmad, Afeefa Khadija, Syeda Mahvish Zahra, Ashiq Hussain

**Affiliations:** 1 Institute of Food Science and Nutrition, University of Sargodha, Sargodha, Pakistan; 2 Department of Environmental Design, Health and Nutritional Sciences, Allama Iqbal Open University, Islamabad, Pakistan

**Keywords:** Stunting, wasting, creatinine, genetics, dietary factors

## Abstract

**Background:**

Across the globe, 149 and 49 million children are stunted and wasted, respectively. Prevalence of stunting and wasting is 40.2 and 17.7% in children of Pakistan. Stunting and wasting are accompanied with genetics, dietary factor, lack of information, bottle feeding, illiterate parents, less birth interval, infection such as diarrhoea, low birth weight, mother suffering from malnutrition during pregnancy, breastfeeding, pharmaceutical, and hormonal, psychosocial, and low social-economic status.

**Methods:**

Stunted and wasted children and their mothers were called on and detail discussions related to research plan were carried out. Informed consent was assured from mothers, for participation in the study. The demographics, anthropometrics, vital signs observations, body composition, clinical signs and symptoms, dietary intake and associated biomarkers (CBC, level of urea nitrogen in blood, serum albumin globulin and serum creatinine.) were tools for nutritional health status assessment. SPSS software was implied on data.

**Results:**

The study found that 27.2% mothers were belonged to stunted children, 17.3% belonged to wasted children, and 50.9% belonged to those children who were suffering from both stunting and wasting condition.57.9% mothers who were illiterate belong to stunted and wasted children. CBC and Hb test was prominent, stunted and wasted children had Hb 9.88 mg/dL, whereas, their mothers had 10.8 mg/dL as average Hb. The average height and weight of stunted and wasted children was 68.6 cm and 7.11 kg respectively. Dietary patterns and diet quality of both mothers and children were poor, due to lack of affordability; they were not able to eat healthy food.

**Conclusion:**

Stunting and wasting ultimately resulted in poor growth and development of children. Most of children were anemic, they height and weight less than WHO growth standards. They had less knowledge and poor intake of food diet pattern so children growth was poor.

## Introduction

Stunting defined as “Moderate and severe - below minus two standard deviations from median height for age of reference population” [44]. Children who are small according to their age are denotes to stunting. Complements of stunted growth are irreversible physical and cognitive damage from which children might be suffering severely. Stunting can affect for late a lifetime and even it has shocking effect on next generation [11].

Wasting defined as “Moderate and severe - below minus two standard deviations from median weight for height of reference population” [44]. Children who are too thin according to their height are denotes to be wasting. Contemporary quick weight loss or the failure to gain weight results in wasting. Moderate and severe wasted children have an increased mortality rate. However, the treatment and cure are conceivable [11].

In 2015, it was globally estimated that 156 million children were stunted, and 50 million children were wasted [45]. Approximately, 8 lac children died due to wasting and more than 1 million children died because of stunting each year [8]. Globally, in 2018, stunted children were 149 million (21.9%) and 49 million (7.3%) of children having age of <05 years were wasted [47]. According to national nutritional survey 2018, 40.2% children were stunted, and 17.7% children were wasted [38].

As compared to girls, stunting and underweight is more common in boys. Causative factors that are associated with poor health and nutrition of children are low socioeconomic status, huge family size, lack of knowledge [5]. Children are 8 and 10% stunted and wasted respectively. Considerably, wasting and stunting are not link related with gender [37]. Stunting is linked birth interval less than two years and with civilization [16]. Children of those fathers who are working as public employer, agriculturalists and salespersons are more stunting as associate with property-owners [26].

For dietary modification nutritional health status assessment is a crucial point. Nutritional health status assessment includes evaluating information related to demographics, anthropometric measurement, biochemical assessment, clinical health manifestation, and eating habits [29, 46]. The nutrients requirement or consumption balance in a state of symmetry to sustained nutritional health. Nutritional health status assessment is 1st stage to design health approach in public to fight malnutrition. Malnutrition transpires when total consumption of nutrient is decreased than requirements. Malnutrition chiefs to sequence of physiological fluctuations, metabolic anomalies, decreases tissue and organ function and damage to body mass. The main purpose of such types of assessments is to find out magnitude, types and distribution of malnutrition in diverse geomorphologic regions to recognize risked group and govern contributing aspect [28].

The components of nutritional health status assessment of an individual are anthropometric measurements, biochemical evaluation, clinical examination and dietary assessment. The scientific measurement of individual body is known as anthropometric. The method which is used for nutritional assessment of individual is very quick, simple, easy and inexpensive. Height, weight, mid upper arm circumference (MUAC), head and chest circumferences (only in young and infant children) and skin fold thickness. The skilled non-medical and medical staff can assemble data related to anthropometry. Weight of a person would be documented in kilograms. The measuring weight individual should be wear minimum clothing and without shoes. Weight interpretation in children is more reliant on age of child. As compared to spring scales beam scale is more consistent for documentation of weight. For children under 2-years of age special baby scales are required because they provide precise measurement of weight [35].

In meta-analysis-based study it was report that if pre-pregnant woman had BMI below 20 kg/m ^2^ was associated with a pointedly more hazard for intrauterine growth restriction (IUGR). In developed countries, it has been predictable poor nutritional status of mothers during pregnancy lead to 14% of fetuses with IUGR and further maternal stunting lead to 18.5% in their offsprings [1].”

## Objectives of study

- Evaluating about dietary habits in children and their mothers
- Evaluated nutritional status of stunted and wasted children and their mother
- Find out the prevalence of stunting and wasting volunteers

## Limitation

- Signs and symptoms might be taking long time to develop, due which it become difficult to link it with diet and nutritional health status
- Many signs and symptoms are not specific for stunting and wasted
- Due to lack of skinfold caliper, during research I had made use of veriner caliper

## Material and methods

### Research design

The study design was randomized controlled clinical trial (MacMahon and Trichopoulos 1996). According to this design, firstly I had selected children of age group 6 to 60 months and their mothers, because stunting and wasting condition found in children of that age group. To found association of stunted and wasted in children and their mothers I had assessed nutritional health status of children and their mothers. Assessment consisted of four parts i.e., demographics, biomarkers, anthropometrics, and food frequency questionnaire.

### Approach and informed consent

The target population was approached; the research project was explained and discussed in detail. The information, education and communication (IEC) materials were provided to mothers. The informed consent was taken from the parents of the children who were part of research project (Martinez and Gonzalez [22]).

### Demographics for selection of children

The demographic data of the agreed parents was taken on pre-designed Performa of questionnaires. The children and their mothers who meet the said criteria were selected for further studies.

### Biomarker of selected children

The blood samples of selected children were collected by hospital lab in charge in coded and specified blood (1-2 cc) collecting containers for determination of CBC, serum albumin, serum globulin, serum creatinine, and level of urea nitrogen in blood to fulfill the criteria of inclusion and exclusion as mentioned earlier.

### Study population

The ethically inform consent was taken from Punjabi mothers of children. Children below 5 years aged and the sample size of my study was 57 for stunted children and 25 for wasted children were screening out for stunting and wasting and their mothers (18–30 and 30-45 years) were also assessed to find relationship between stunted and wasted children and their mothers and they belong to low socio-economic status for study by method as adapted by [36].

### Study location

Primary and Secondary Healthcare Department DHQ hospital, Hafizabad was selected as study site for the research work after getting written permission from the competent authorities. The families belong to Rular, Pri-urban, urban area (adapted from: [17]).

### Sampling technique

The stunted and wasted children and their mothers were the target population. The selection of the children was carried out in according with two Stage Sampling that is convenience sampling technique; which a part of Non-Probability Sampling method as described by Muhammad [36].

### Data collection and analysis

Data was presented with the help of tables, charts, and graphs where necessary. Data collected was analyzed with the help of statistical software (SPSS). Appropriate statistical tests were used. Both parametric and non-parametric tests were used for testing significance. Descriptive statistics were run to check the distribution and frequency of data. Analysis of variance and post hock LSD was applied to seek the level of significance.

### Permission

The written permission from mothers of Dist. Hafizabad was taken to collect data from them.

### Selection procedure for volunteers

The children and their mothers were screened out for stunted and wasted, selected stunted and wasted for study by method as Adapted by Mahmood and Butt [32].

### Ethical consideration

The research proposal was approved by Biosafety and Ethical Review Committee, University of Sargodha, Sargodha (Adapted from Gibney [19]).

### Sample size

The sample size for stunted and wasted children and their mothers were calculated by the following formula; considering estimated prevalence of variable of interest (stunted and wasted), required level of confidence (95%) and 5% margin of error (adapted by [31]).

#### Step 1. Base sample size calculation (n)

$$\mathbf{n}=\frac{{\mathbf{t}}^{\mathbf{2}}\times \mathbf{p}\left(\mathbf{1}-\mathbf{p}\right)}{{\mathbf{m}}^{\mathbf{2}}}$$

Where,

n = required sample size.

t = confidence level at 95% (standard value of 1.96).

p = estimated/reported prevalence of stunted and wasted children among children of age group 6 to 60 months in Pakistan.

m = margin of error at 5% (standard value of 0.05).

#### Step 2: Design effect (D)

To minimize the design effect; the sample size was multiplied by 2 that is considered as D for nutritional studies.

#### Step 3: Contingency (Final Sample Size)

Final sample size was calculated by given formula.

n_final_ = (n x D) + 5 % of (n x D)

Where,

n _final_ = final sample size.

n x D = sample size with design effect.

% = the calculation and addition of 5% accounted for contingencies which were represent no response, drop out and data recording errors

So, the sample size of my study was 57 for stunted children and 25 for wasted children.

### Anthropometrics and nutritional status assessment

#### Energetic of children

The anthropometric measurements of the selected children like height, weight, body composition, head circumference, neck circumference, chest circumference, waist circumference, MUAC, caloric intake and caloric requirements were calculated to assess their nutritional status.

In children, body fat was measured by using Vernier Caliper, measurement of body fat was taken from sites of biceps, triceps, suprailiac, and subscapular (adapted from: [10]).

For assessment of mothers, weight, body fat, muscle mass, body water, bone mass, AMR, and BMR were calculated through BF-105 (Burer, Germany). The obtained data was compared with the standards for the evaluation of the nutritional and health status of the mothers (adapted from: Jana [23]).

#### Dietary intakes assessment

Performa of dietary intakes was filled up of volunteers collected by the researcher. The dietary intakes of volunteers were assessed through Food Guide Pyramid based FFQ at baseline (quantitative base question) (adapted from: Gibson [20]).

#### Clinical assessment

Clinical signs and symptoms related to stunting and wasting were observed in the children under study. All the information and data were used to find out the correlation between nutritional status and stunting and wasting. (Tables 3, 4, 5, 6).

#### Medical history

Medical history of the stunted and wasted children, and their mothers, about the prevalence of stunted and wasted was explored. (Table 2).

#### Research instruments

a) Questionnaire: For screening/nutritional assessment of anemia.

b) Biochemical tests: CBC, level of urea nitrogen in blood, serum globulin, serum albumin, serum creatinine.

#### Delimitations and limitations

The target population, study locale, experts and researcher were available (delimitations) while social set up, finance, logistics, lack of availability of skinfold caliper and laboratory investigations were the constraints faced during the research work (Adapted from [12]).

## Results and discussion

The basic purpose of study is find association of stunted and wasted children and their mothers for their nutritional health status. There is large number of stunted and wasted children age of 6 months to 5 years age. Those children who were part of study having religion Islam, Pakistani nationality, and their mothers were housewife. The basic factors of stunted and wasted children are poor diet. In study children whose age is between 6 months to 5 years and their mothers were included in nutritional assessment. Nutritional health status assessment includes demographics of mothers, biomarkers of children, biomarkers of mothers, anthropometric measurement of stunted and wasted children and their mothers, FFQ of mothers and FFQ of children were divided in two groups according to their age group i.e., in 1st group FFQ of children from 6 to 24 months were included, whereas in 2nd group children whose age is from 2 to 5 years were included. The observed results mentioned below.

### Demographics of mothers

Table 1 showed that all mothers who come with children in hospital were Pakistani. The prevalence of stunted, wasted, and stunted and wasted children was 27.17, 17.34 and 50.87%, respectively. Majority of mothers having stunted and wasted children which are about 60.0% belonged to age group of is 30–45 years while 47.07% mothers having stunted and wasted children belonged to age group of 18–30 years. It is evident that children of those mothers of age group 30–45, were more prone to be stunted and wasted. In a study it was observed that if age of mother is more than 18 years and visit parental care clinic more than 3 times during pregnancy, children of those mothers were likely to less stunted [24]. In some studies it was observed that most of mothers with stunted and wasted children mean average rang of age were 30.3 ± 6.0 years about 75.3% with stunted and wasted children [3]. Children whose mothers lived in rural areas were more likely to have stunted and wasted children i.e. 57.6%, whereas those mothers who lived in urban areas 44.1% belonged to stunted and wasted children. Majority of mothers of those children who were likely to be more stunted and wasted lived in rural areas i.e. 56.7% [24]. In a dissimilar study, it was observed in Indonesia that children who lived in rural areas were less prone to be stunted as compared to those children who lived in urban area [48]. In a similar study, prevalence of stunting and wasting higher in rural areas as compared to urban areas [4]. Mothers who lived nuclear family system, i.e. 60.5% belonged to stunted and wasted children on the other hand 41.4% mothers who come to hospital were lived in joined family system, belonged to stunted and wasted children. In a similar study, it was observed that the children of nuclear family more likely to be stunted and wasted [25]. In another similar study, which was conducted in Pakistan, results showed that stunting and wasting mostly prevalent among children of nuclear family system as compared to joint family system (Mahmood et al. [33]). It was revealed that mothers who had sedentary physical activity were belonged to 50% stunted and wasted children, whereas those mothers who had light active physical activity belonged to 51.2% stunted and wasted children. In a study, it was indicated mothers who did not work had less significant proportions of stunting i.e. 43.4% in contrast to mothers who work for the same period that was 49.8%, whereas proportion of wasting was significant in those mothers who did not work in contrast to those mothers who worked [27].

**Table 1:** Demographics of mothers

		Nutritional status				Total
		Stunted	Wasted	Stunted Wasted	Normal	
Nationality	Pakistani	27.2%	17.3%	50.9%	4.6%	100.0%
Age	18–30	28.1%	18.8%	47.7%	5.5%	100.0%
	30–45	24.4%	13.3%	60.0%	2.2%	100.0%
Total		27.2%	17.3%	50.9%	4.6%	100.0%
Type of residence	Rural	21.7%	18.5%	57.6%	2.2%	100.0%
	Peri-urban	38.5%	23.1%	38.5%	0.0%	100.0%
	Urban	32.4%	14.7%	44.1%	8.8%	100.0%
Total		27.2%	17.3%	50.9%	4.6%	100.0%
Family system	Joint	32.2%	19.5%	41.4%	6.9%	100.0%
	Nuclear	22.1%	15.1%	60.5%	2.3%	100.0%
Total		27.2%	17.3%	50.9%	4.6%	100.0%
Physical activity	Sedentary	26.1%	19.6%	50.0%	4.3%	100.0%
	Light active	27.6%	16.5%	51.2%	4.7%	100.0%
Total		27.2%	17.3%	50.9%	4.6%	100.0%

### Biomarkers of children

From Table 2, it was revealed that results were highly significant, the level of all components of blood were decreased in stunted and wasted children. Hb is most important component of blood, its decrease level show that children were anemic. Hb of normal children was 12.59 (mg/dL), stunted children Hb was 11.8 (mg/dL), wasted children Hb was 10.85 (mg/dL), and whereas those children who were both stunted and wasted have Hb 9.88 (mg/dL). In a similar study, level of Hb decreased in stunted and wasted children, anemia was observed in them [15]. Low level of Hb was observed in stunted children, prevalence of anemia is higher among stunted children [41]. In other similar study, it was observed that stunted and wasted children anemic [43].

**Table 2:** Biomarkers of Children

Biomarkers	Groups	Value	***P*** values
**Hemoglobin**	Normal	12.59 ± 0.18A	0**
	Stunted	11.8 ± 0.21B	
	Wasted	10.85 ± 0.53C	
	Stunted and Wasted	9.88 ± 0.21D	
**Hematocrit**	Normal	12.59 ± 0.18A	0**
	Stunted	11.8 ± 0.21B	
	Wasted	10.85 ± 0.53C	
	Stunted and Wasted	9.88 ± 0.21D	
**Red Blood cells**	Normal	4.66 ± 0.34A	0**
	Stunted	3.88 ± 0.22B	
	Wasted	3.36 ± 0.15C	
	Stunted and Wasted	2.85 ± 0.16D	
**Mean Corpuscular Volume**	Normal	4.66 ± 0.34A	0**
	Stunted	3.88 ± 0.22B	
	Wasted	3.36 ± 0.15C	
	Stunted and Wasted	2.85 ± 0.16D	
**Mean Corpuscular Hemoglobin**	Normal	27.18 ± 0.82A	0**
	Stunted	25.21 ± 0.58B	
	Wasted	23.76 ± 1.11C	
	Stunted and Wasted	20.13 ± 0.82D	
**Mean Corpuscular Hemoglobin concentration**	Normal	37.17 ± 2.37A	0**
	Stunted	30.33 ± 1.31B	
	Wasted	27.17 ± 1.15C	
	Stunted and Wasted	24.43 ± 1.34D	
**White blood cells**	Normal	13.29 ± 0.2A	0**
	Stunted	12.5 ± 0.25B	
	Wasted	11.55 ± 0.55C	
	Stunted and Wasted	10.58 ± 0.2D	
**Creatinine**	Normal	0.45 ± 0.03A	0**
	Stunted	0.4 ± 0.03B	
	Wasted	0.35 ± 0.03C	
	Stunted and Wasted	0.3 ± 0.03D	
**Serum Albumin**	Normal	5.41 ± 0.32A	0**
	Stunted	5.05 ± 0.3B	
	Wasted	4.53 ± 0.32C	
	Stunted and Wasted	3.99 ± 0.32D	
**Serum Globulin**	Normal	6.12 ± 0.26A	0**
	Stunted	5.61 ± 0.61A	
	Wasted	4.95 ± 0.75B	
	Stunted and Wasted	4.26 ± 0.76C	
**Blood Urea Nitrogen**	Normal	12.75 ± 1.87A	0.558^NS^
	Stunted	13.6 ± 1.75A	
	Wasted	13.27 ± 1.71A	
	Stunted and Wasted	13.27 ± 1.77A	

### Anthropometrics of children

In Table 3, non-significant results were found. It was found that normal children have 63.6 average value of pulse rate, stunted have 62.63 pulse rates, wasted have 60.77 pulse rate, and whereas both stunted and wasted have 57.31 pulse rates. In a similar study, it was observed that stunting and wasting children were at risk of developing congenital heart disease and is associated with anemia and heart failure due to pulse rate of children decreased [6]. Non-significant result was concluded, from it was found children who were normal have 98.58 °F temperature, stunted children have 98.38 °F, wasted children have 98.28 °F, and those children who were both stunted and wasted have 97.87 °F. Non-significant results were observed related to blood oxygen saturation. It was observed that children who were normal have 98.25, stunted have 97.32, wasted have 95.34, and both stunted and wasted children have 94.76 blood oxygen saturation levels. In Table 6, significant results were concluded. So, it was observed that those children who were normal have average height 76.03 cm, stunted children have average height 72.75 cm, wasted children have average height 72.5 cm, whereas those children who were both stunted and wasted have average height 68.6 cm. In a study, it was concluded that children who were stunted and wasted, were at greater risk of linear growth retardation particularly in children aged 12–23 months [42]. Highly significant results were obtained related to weight of children. Children who were normal have 9.41 kg was average weight, stunted children have 9.13 kg was average weight, wasted children have 7.23 kg average weight, and some children who were both stunted and wasted have 7.11 kg average weight. In a study, it was observed that the wasted children were interpreted as those children who have low value of weight for height in that condition muscle and body fat were reduced, that is, the child is wasted [21]. Highly significant value of triceps was obtained. Average value of triceps in normal children 8.22 mm, in stunted children 8.04 mm, in wasted children 6.32 mm, in those children who were both stunted and wasted low value of triceps 6.05mmwas observed. In one study, significant results related to triceps were found, 8.57 mm in girls and 8.15 mm in boys triceps [18]. Non-significant value of biceps was revealed. Average value of biceps was found in normal children have 6.65 mm, in stunted children have 6.56 mm, in children have wasted 6.25 mm, and least average value of biceps observed in both stunted and wasted have 5.65 mm. Highly significant result related to subscapular was observed. Normal children have 8.39 mm, stunted children have 8.3 mm, wasted children have 6.22 mm, and whereas those children who were both stunted and wasted have 6.13 mm average value of subscapular. In a similar study highly significant result were found, girls had 6.31 mm and boys had 5.98 mm of subscapular [18]. Non-significant results were obtained related to suprailiac. Average value of suprailiac in different group of children such as normal children have 9.09 mm, stunted children have 7.19 mm, wasted children have 6.58 mm, and those children who were both stunted and wasted have 5.79 mm.

**Table 3:** Anthropometric of children

	Groups	Value	***P*** values
**Pulse Rate**	Normal	63.6 ± 2.61A	0.708^NS^
	Stunted	62.63 ± 11.8A	
	Wasted	60.77 ± 4.02A	
	Stunted and Wasted	57.31 ± 4.54A	
**Temperature**	Normal	98.58 ± 0.13A	0.195^NS^
	Stunted	98.38 ± 0.32AB	
	Wasted	98.28 ± 0.19AB	
	Stunted and Wasted	97.87 ± 0.42B	
**Blood Oxygen Saturation**	Normal	98.25 ± 4.02A	0.65 ^NS^
	Stunted	97.32 ± 1.99A	
	Wasted	95.34 ± 2.06A	
	Stunted and Wasted	94.76 ± 1.32A	
**Height (cm)**	Normal	76.03 ± 1.41A	0.001*
	Stunted	72.75 ± 2.26AB	
	Wasted	72.5 ± 2.39AB	
	Stunted and Wasted	68.6 ± 0.98B	
**Weight (kg)**	Normal	9.41 ± 0.37A	0**
	Stunted	9.13 ± 0.6A	
	Wasted	7.23 ± 0.38B	
	Stunted and Wasted	7.11 ± 0.22B	
**Triceps (mm)**	Normal	8.22 ± 0.2A	0**
	Stunted	8.04 ± 0.58A	
	Wasted	6.32 ± 0.36B	
	Stunted and Wasted	6.05 ± 0.22B	
**Biceps (mm)**	Normal	6.65 ± 0.26A	0.884 ^NS^
	Stunted	6.56 ± 0.69A	
	Wasted	6.25 ± 0.9A	
	Stunted and Wasted	5.65 ± 0.3A	
**Subscapular (mm)**	Normal	8.39 ± 0.21A	0**
	Stunted	8.3 ± 0.72A	
	Wasted	6.13 ± 0.34B	
	Stunted and Wasted	6.22 ± 0.21B	
**Suprailiac (mm)**	Normal	43.63 ± 1.08A	0.133^NS^
	Stunted	41.57 ± 0.59B	
	Wasted	39.84 ± 0.76 BC	
	Stunted and Wasted	39.16 ± 0.38C	

From Table 4, highly significant results were found about waist circumference of children. Average value of waist circumference that observed in normal children group has 43.18 cm, group of stunted children have 42.25 cm, group of wasted children have 39.5 cm, and group of those children who were both stunted and wasted have 39.26 cm. In a study, dissimilar results were found, in most of children waist circumference of children was normal [34]. Average value of MUAC that had been observed in group of normal children had 13.91 cm, stunted children had 13.74 cm, wasted children had 11.69 cm, and group of those children who were both stunted and wasted had 11.45 cm. in a similar study similar result were found, MUAC of children was low, who were stunted wasted and underweight [39]. In a study similar most significant results were found, children who were wasted had MUAC less than 11.5 cm [7]. It was observed that normal children have 44.5 cm, stunted children have 44.27 cm, wasted children have 42.87 cm, and a group of those children who were both stunted and wasted have 42.14 cm average value of head circumference. In a similar study, significant results of head circumference were obtained [18]. In a similar study, significant results related head circumference were obtain that stunted children had 44.67 cm and waste children had 48.54 cm head circumference [40]. Group of normal children have 43.63 cm chest circumference, group of stunted children had average value of chest circumference 41.57 cm, group of wasted children have average value of chest circumference that was 39.84 cm, and in group of those children who were both stunted and wasted had 39.16 cm average value of chest circumference. In a study, non-significant result were revealed, stunted children had 49.17 cm and wasted children and 49.79 cm chest circumference [40]. Neck circumference of children who were normal had 24.73 cm, stunted had 23.86 cm, wasted had 23.17 cm, and group of both stunted and wasted had 21.4 cm average value.

**Table 4:** Anthropometric of children

	Groups	Value	***P*** values
**Waist Circumference (cm)**	Normal	43.18 ± 0.49A	0.708^NS^
	Stunted	42.25 ± 1.19AB	
	Wasted	39.5 ± 0.72 BC	
	Stunted and Wasted	39.26 ± 0.46C	
**MUAC (cm)**	Normal	13.91 ± 0.3A	0**
	Stunted	13.74 ± 0.1A	
	Wasted	11.69 ± 0.19B	
	Stunted and Wasted	11.45 ± 0.12B	
**Head Circumference (cm)**	Normal	44.5 ± 0.57AB	0**
	Stunted	44.27 ± 0.31A	
	Wasted	42.87 ± 0.4 BC	
	Stunted and Wasted	42.14 ± 0.28C	
**Chest Circumferenc (cm)**	Normal	43.63 ± 1.08A	0**
	Stunted	41.57 ± 0.59B	
	Wasted	39.84 ± 0.76 BC	
	Stunted and Wasted	39.16 ± 0.38C	
**Neck Circumference (cm)**	Normal	24.73 ± 0.29A	0**
	Stunted	23.86 ± 0.46AB	
	Wasted	23.17 ± 0.18B	
	Stunted and Wasted	21.4 ± 0.27C	

### Anthropometric of mothers

In Table 5, non-significant result was revealed. Mothers of normal children average pulse rate were 96.13, those of stunted children’s mother have 92.87 average pulse rate, mothers of wasted children average pulse rate were 90.26, and whereas it was observed that mothers of stunted and wasted average pulse rate was 88.68. Observations were made that non-significant results were revealed related to body temperature of mothers. Mothers of normal children have 98.4 °F, mothers of stunted children have 98.3 °F, and mothers of wasted children have 98.19 °F, and while, mothers of stunted and wasted children have 98.06 °F average body temperature. Non-significant results were observed related to blood oxygen saturation, Blood oxygen saturation of mothers, who have normal children was 97.79, mother of stunted children has 97.53, mothers of wasted have 97.4, and mothers of both stunted and wasted have 97.13. In Table 8, non-significant results about height were observed, mothers had average height of normal children had 152.17 cm, while, mothers of stunted children had 151.27 cm, whereas mothers of wasted had 151.15 cm and mothers of both stunted and wasted 150.25 cm respectively. In a dissimilar study, significant result related to height of mothers, it was observed in that study that mothers whose were short statures <145 cm height were more likely to had stunted children [24]. In another study, it was revealed that those mothers have height less than 150.1 cm were more like to have stunted children [2]. In one study, it was observed that height of mothers showed negative relation with stunted but not wasted children [3]. Non-significant results were obtained about weight of mothers, mothers had average weight of normal children had 64.55 kg, while, mothers of stunted children had 61.67 kg, whereas mothers of wasted had 60.46 kg, and mothers of both stunted and wasted 57.72 kg respectively. Body fat of mothers was showed non-significant results, mothers’ body composition was assessed, through assessment body fat, was observed, from which mothers of normal children had 34.39%, mothers of stunted children, 34.74%, mothers of wasted children had 35.75%, and, mothers of both stunted and wasted had 37.38%. Non-significant result about body water in mothers was found, mothers of normal children had 44.66% body water, stunted children mothers had 43.74% body water, mothers of wasted children had 43.65% body water, and mothers of both stunted and wasted had 42.54% body water. Non-significant results were revealed about the muscle mass of mothers. Muscle mass was measured of mothers by BF105 machine, mothers of normal children had 31.58%, mothers of stunted children had 31.17%, mothers of wasted children had 30.89%, and mothers of both stunted and wasted children had 31.49%. Non-significant results were found about bone mass of mothers. In Table 8, mothers of normal children have 9.44% bone mass, mothers of stunted children have 9.33% bone mass, mothers of wasted children have 9.33% bone mass, and mothers of stunted and wasted children have 9.19% bone mass.

**Table 5:** Anthropometric of mothers

	Groups	Value	***P*** values
**Pulse Rate**	Normal	96 ± 13.99A	0.321 ^NS^
	Stunted	92.87 ± 13.8A	
	Wasted	90.26 ± 15A	
	Stunted and Wasted	88.68 ± 13.41A	
**Temperature**	Normal	98.4 ± 0.77A	0.166 ^NS^
	Stunted	98.3 ± 0.66A	
	Wasted	98.19 ± 0.33A	
	Stunted and Wasted	98.06 ± 0.18A	
**Blood Oxygen Saturation**	Normal	97.79 ± 1.46A	0.465 ^NS^
	Stunted	97.53 ± 1.3A	
	Wasted	97.4 ± 1.4A	
	Stunted and Wasted	97.13 ± 1.36A	
**Height (cm)**	Normal	152.17 ± 5.24A	0.692 ^NS^
	Stunted	151.27 ± 8.42A	
	Wasted	151.15 ± 6.05A	
	Stunted and Wasted	150.25 ± 6.16A	
**Weight (kg)**	Normal	64.55 ± 8.31A	0.395 ^NS^
	Stunted	61.67 ± 12.51A	
	Wasted	60.46 ± 11.48A	
	Stunted and Wasted	57.72 ± 13.04A	
**Body Fat (%)**	Normal	34.39 ± 4.42A	0.298
	Stunted	34.74 ± 4.96A	
	Wasted	35.75 ± 5.19A	
	Stunted and Wasted	37.38 ± 2.75A	
**Body Water (%)**	Normal	44.66 ± 4.59A	0.627 ^NS^
	Stunted	43.74 ± 4.76A	
	Wasted	43.65 ± 4.64A	
	Stunted and Wasted	42.54 ± 3.05A	
**Muscle Mass (%)**	Normal	30.29 ± 1.88A	0.815 ^NS^
	Stunted	31.57 ± 4.03A	
	Wasted	31.58 ± 2.84A	
	Stunted and Wasted	31.49 ± 3.65A	
**Bone Mass (%)**	Normal	9.44 ± 0.41A	0.487 ^NS^
	Stunted	9.33 ± 0.49A	
	Wasted	9.33 ± 0.44A	
	Stunted and Wasted	9.19 ± 0.62A	

In Table 6, non-significant results were obtained, mothers of healthy children AMR were assessed, whereas mothers of normal children had 1676.75, mothers of stunted children had 1650.94, mothers of wasted had 1627.01 mothers of both stunted and wasted had 1579.93. Mothers BMR was assessed, whereas mothers of normal children had 1368.23, mothers of stunted children had 1317.75, mothers of wasted had 1267.62 and mothers of both stunted and wasted had 1238.23. Head circumference of mothers were revealed, mothers of normal children had 52.07 cm head circumference, stunted children’s mothers had 52.92 cm, those of wasted children’s mothers had 52.92 cm, and while mother of both stunted and wasted had 52.5 cm. In Table 6, non-significant results were disclosed about waist circumference of mothers, mothers of normal children had 95.63 cm waist circumference, mothers of stunted children had 92.55 cm waist circumference, mothers of wasted children had 91.87 cm waist circumference, and mothers of both stunted and wasted mothers had 91.63 cm waist circumference. In a study dissimilar result was revealed that mothers’ waist circumference show positive relationship with their children who were stunted [14]. Non-significant result was divulging related to MUAC, mothers of healthy children had 27.35 cm MUAC, mothers of stunted children had 26.08 cm MUAC, mothers of wasted children had 25.88 cm MUAC, and mothers of both stunted and wasted had 25.36 cm MUAC. In a study, MUAC of mothers who were belonged to stunted and wasted children had less than 23.5 cm [40]. It was founded that mothers of normal children had 89.88 cm, stunted children’s mothers had 87.65 cm, mothers of wasted children had 86.65 cm, and on the other hand, mothers of both stunted and wasted mothers had 86.4 cm. Neck circumference of mothers was measured, mothers of normal children had 32.89 cm, stunted children’s mothers had 32.68 cm, wasted children’s mothers had 32.16 cm, and mothers of both stunted and mothers had 31.55 cm.

**Table 6:** Anthropometric of mothers

	Groups	Value	***P*** values
**AMR**	Normal	1676.75 ± 150.9A	0.379 ^NS^
	Stunted	1650.94 ± 190.3A	
	Wasted	1627.01 ± 188.2A	
	Stunted and Wasted	1579.93 ± 208.1A	
**BMR**	Normal	1368.23 ± 1059A	0.824 ^NS^
	Stunted	1317.75 ± 99.1A	
	Wasted	1267.62 ± 139.7A	
	Stunted and Wasted	1238.23 ± 163.5A	
**Waist Circumference (cm)**	Normal	95.63 ± 6.16A	0.786 ^NS^
	Stunted	91.87 ± 9.87A	
	Wasted	91.63 ± 12.87A	
	Stunted and Wasted	92.55 ± 10.09A	
**MUAC (cm)**	Normal	27.35 ± 2.39A	0.433^NS^
	Stunted	26.08 ± 3.11A	
	Wasted	25.88 ± 3.04A	
	Stunted and Wasted	25.36 ± 3.58A	
**Head Circumference (cm)**	Normal	53.07 ± 1.85A	0.867 ^NS^
	Stunted	52.92 ± 2.09A	
	Wasted	52.92 ± 1.7A	
	Stunted and Wasted	52.5 ± 1.58A	
**Chest Circumference (cm)**	Normal	89.88 ± 7.38A	0.806^NS^
	Stunted	87.65 ± 11.63A	
	Wasted	86.65 ± 9.67A	
	Stunted and Wasted	86.4 ± 11.35A	
**Neck Circumference (cm)**	Normal	32.89 ± 4.96A	0.261^NS^
	Stunted	32.68 ± 1.98A	
	Wasted	32.16 ± 2.56A	
	Stunted and Wasted	31.55 ± 2.76A	

### Food frequency questionnaire of children

To find better result related to food intake of children, they were divided into two groups according to their age i.e. 6–24 months and 24 to 60 months. The intake of children whose age below than 24 months as compared to those whose age is more than 24 months. From Table 7: it concluded that intake of all food groups were less in children of age group (6–2 months), they take 2.1 serving per week of cereals, 2.74 servings per week of fruit, 0.47 servings per week of vegetables, 0.66 servings per week of meat group, 68.86 servings per week of milk, 0.34 servings per week of fat & oil, 4.78 servings per week of junk/snack, 0.86 servings per week of miscellaneous, and 0.73 litter per week of water. On the other hand, children belong to age group (24–60 months), were taking more food groups servings per week. They take average 10.41 servings per week of cereals, 7.87 servings per week of fruits, 1.18 servings per week of vegetables, meat 2.87, 53.61 servings per week of milk, fat & oil 3.13 servings per week, junk/snack 8.6 servings per week, miscellaneous 6.73 servings per week, and 1.75 l per week of water. In one study, it was found about food that consumption of starchy staples, legumes, and oil was common. Whereas, intake of fruits and vegetables were less in children. About half children were consumed dairy products. Intake of egg and fish was also limited [13]. In other study, they also divided the children in two group i.e. 6–24 and 24–60 months, it was revealed that intake of children, that they daily consumed less amount of vegetables and meat but high intake of soft drink, fried food salty snack, and sweets [9].

**Table 7:** FFQ of children

Food group	Children (6–24)	Children (24–60)
**Cereals**	2.1 ± 2.65B	10.41 ± 6.45A
**Fruits**	2.74 ± 3.78B	7.87 ± 14.47A
**Vegetable**	0.47 ± 0.85B	1.18 ± 1.53A
**Meat**	0.66 ± 1.49B	2.87 ± 2.78A
**Milk**	68.86 ± 35.26A	53.61 ± 53.1A
**Fat & oil**	0.34 ± 1.37B	3.13 ± 4.02A
**Junk/Snack**	4.78 ± 6.3B	8.6 ± 7.42A
**Miscellaneous**	0.86 ± 2.29B	6.73 ± 6.99A
**Water (L)**	0.73 ± 0.69B	1.75 ± 0.71A

### Food frequency questionnaire of mothers

In Table 8, non-significant results were obtained related to intake of cereals, it was concluded that 63.13 servings per week of cereals were taken by mothers of children who were stunted and wasted, they were take less serving of cereals as compared to mothers of healthy children i.e. 72.5 servings per week. It was observed that all mothers were taking more 6 servings per day of cereals either they belong to mothers of stunted and wasted children or mothers of healthy children. Non-significant results were observed related to intake of fruits servings, it was concluded that least 9.5 servings per week of fruits were taken by those mothers of children who were both stunted and wasted, they were taking less servings as compared to recommended servings of fruits, for healthy persons it is recommended that they should take 2–4 servings of fruits on daily basis. While mothers of healthy children were taking 16.81 servings per week of fruits, they are taking more than 2 servings of fruits. So it was concluded that mothers of stunted and wasted taking less fruits due to lack of affordability of fruit due to which nutritional status of children was also affected. It was disclosed that less 15.25 servings per week of vegetables were taken by mothers of stunted and wasted children. They were taking less than 3–5 servings per day of vegetables that recommended according to food pyramid, whereas mothers of healthy children were taking 24.67 servings per week of vegetables, they were taking more than 3 servings per day of vegetables, so it showed that less intake of vegetables by mothers had its effect on the health of children. It was observed that mothers of stunted and wasted children had least intake of meat i.e., 5.73 servings per week as compare to mothers of healthy children i.e. 7.5 servings per week. From study it was concluded that mothers of all children were taking less than recommended servings of meat group i.e. 2–3 servings per day. Results related to intake of milk showed variations, it can be concluded that mothers of stunted and wasted children have least 7.88 servings of milk per week as to compare to mothers of healthy children i.e. 12.77. It was revealed that mothers of stunted and wasted children were taking less 2 servings of milk per day which also affected the nutritional status of children. It was observed that mothers of stunted and wasted children had high 18.17 servings per week of fat and oil intake as compared to mothers of healthy children, whose intake was 12.75 servings per week. Sparingly used of fat and oil is recommended but their intake of fat and oil was increased. This also affected the nutritional status of children. It was concluded that highest 26.98 servings per week of junk and snack were observed in mothers of stunted and wasted children but zero serving of junk and snack was recommended. So this showed that all mothers had intake of junk and snack food group, this showed negative impact on the health of children. Non-significant results were concluded related to miscellaneous food intake, it was observed that highest 1.75 servings per week of miscellaneous food were taken by mothers of stunted and wasted children. Fewer servings were taken by mothers of healthy children. Non-significant results were concluded related to water intake, mothers of children who were stunted and wasted taken 7.19 L per week; they had least intake of water than mothers of healthy children.

**Table 8:** FFQ of mothers

Food group	Groups	Value	***P*** values
**Cereals**	Normal	72.5 ± 2.44A	0.494^NS^
	Stunted	71 ± 2.2A	
	Wasted	64.27 ± 1.12A	
	Stunted and Wasted	63.13 ± 2.21A	
**Fruits**	Normal	16.81 ± 1.69A	0.483^NS^
	Stunted	13.23 ± 1.71A	
	Wasted	12.37 ± 1.38A	
	Stunted and Wasted	9.5 ± 1.54A	
**Vegetable**	Normal	24.67 ± 1.01A	0.262^NS^
	Stunted	20.26 ± 1.09AB	
	Wasted	18.56 ± 1.98AB	
	Stunted and Wasted	15.25 ± 1.76B	
**Meat**	Normal	7.5 ± 1.43A	0.823^NS^
	Stunted	6.42 ± 1.09A	
	Wasted	6.23 ± 1.05A	
	Stunted and Wasted	5.73 ± 1.19A	
**Milk**	Normal	12.77 ± 1.58A	0.995^NS^
	Stunted	10.69 ± 1.58AB	
	Wasted	9.13 ± 1.2AB	
	Stunted and Wasted	7.88 ± 1.49B	
**Fat and oil**	Normal	12.75 ± 1.89B	0.208^NS^
	Stunted	17.27 ± 1.62AB	
	Wasted	17.69 ± 1.31AB	
	Stunted and Wasted	18.17 ± 1.19A	
**Junk/ snack**	Normal	13.5 ± 1.43AB	0.093^NS^
	Stunted	18.71 ± 1.28B	
	Wasted	23.4 ± 1.89AB	
	Stunted and Wasted	26.98 ± 1.8A	
**Miscellaneous food**	Normal	0.48 ± 0.01A	0.372^NS^
	Stunted	0.6 ± 0.29A	
	Wasted	0.87 ± 0.17A	
	Stunted and Wasted	1.75 ± 0.26A	
**Water**	Normal	12.67 ± 1.24A	0.325^NS^
	Stunted	9.91 ± 1.03AB	
	Wasted	9.39 ± 1.25AB	
	Stunted and Wasted	7.19 ± 1.37B	

## Conclusion

From the result of present study, it was revealed that 27.17% children were stunted, 17.34% children were wasted, and 50.87% children were stunted and wasted. Stunting and wasting at this stage ultimately result in poor growth and development of children. Most of children were anemic, their height and weight were less than WHO growth standards. Intake of food was observed in children and mothers that they had poor diet pattern. Due to lack of maternal education, low socio-economic status, poor diet, and lack of awareness about healthy and balanced diet, children faced wasting and stunting. Government should take serious steps concerning with stunting and wasting health issues for the prosperous and healthy nation.

## Data Availability

There are no additional data sets, yet required data collected through questionnaires was collected and has been represented in research article after statistical analyses.

## Abbreviations

FFQ: Food frequency questionnaire
AMR: Active metabolic rate
BMR: Basal metabolic rate
